# Geographical distribution for malignant neoplasm of the pancreas in relation to selected climatic factors in Japan

**DOI:** 10.1186/1476-072X-6-34

**Published:** 2007-07-26

**Authors:** Setsuko Kinoshita, Yukiko Wagatsuma, Masafumi Okada

**Affiliations:** 1 Department of Epidemiology, Graduate School of Comprehensive Human Sciences, University of Tsukuba, Ibaraki, Japan

## Abstract

**Background:**

Malignant neoplasm of the pancreas has become one of the leading causes of death from malignant neoplasm in Japan (the 5th in 2003). Although smoking is believed to be a risk factor, other risk factors remain unclear. Mortality from malignant neoplasm of the pancreas tends to be higher in northern Japan and in northern European countries. A recent study reported that standardized mortality ratios (SMRs) for malignant neoplasm of the pancreas were negatively correlated to global solar radiation level. People residing in regions with lower solar radiation and lower temperatures may be at higher risk of development of malignant neoplasm of the pancreas. Therefore, this study aimed to examine the relationship between SMRs for malignant neoplasm of the pancreas and climatic factors, such as the amount of global solar radiation and the daily maximum temperature in Japan.

**Results:**

The study used multiple linear regression models. Number of deaths and demographic data from 1998 to 2002 were used for the calculation of SMR. We employed mesh climatic data for Japan published in 2006 by the Japan Meteorological Agency. Regression coefficients for the amount of global solar radiation and the daily maximum temperature in males were -4.35 (p = 0.00034) and -2.81 (p < 0.0001) respectively, and those in females were -5.02 (p < 0.0001) and -1.87 (p < 0.0001) respectively. Increased amount of global solar radiation or daily maximum temperature was significantly related to the decreased SMRs for malignant neoplasm of the pancreas in both males and females.

**Conclusion:**

This study suggested that low solar radiation and low temperature might relate to the increasing risk of malignant neoplasm of the pancreas. Use of group data has a limitation in the case of evaluating environmental factors affecting health, since the impact of climatic factors on the human body varies according to individual lifestyles and occupations. Use of geographical mesh climatic data, however, proved useful as an index of risk or beneficial factors in a small study area. Further research using individual data is necessary to elucidate the relationship between climatic factors and the development of malignant neoplasm of the pancreas.

## Background

Malignant neoplasm of the pancreas is associated with a high fatality rate and has become one of the leading causes of death from malignant neoplasm in Japan [1]. Elderly persons who were born during the period 1890–1930 (birth cohort for the beginning of the 20th century) have a higher risk than younger generations. Although the age effect increases similarly for both sexes, the cohort effect is higher in males than in females [2]. The incidence of malignant neoplasm of the pancreas and associated mortality are almost equal due to poor prognosis [3]. Although certain dietary factors such as cholesterol, fat, alcohol and coffee were suspected risk factors [4], recent cohort studies do not support the relationship between malignant neoplasm of the pancreas and these dietary factors [5].

Smoking has been regarded as a risk factor [2], the cessation of which decreases the risk of developing malignant neoplasm of the pancreas [6]. Although cigarette smoking is believed to be a risk factor, it explains only one quarter of the causes of death [7]. An individual's medical history is thought to influence the development of malignant neoplasm of the pancreas. Silverman et al. [8] conducted a case-control study and showed a significantly increased risk associated with diabetes mellitus, cholecystectomy and familial history of malignant neoplasm. The cohort study of Ye et al. [9] demonstrated that diabetes and chronic pancreatitis were associated with higher risk, although an increased risk did not exist for cholecystectomy. Although many studies have dealt with malignant neoplasm of the pancreas in relation to risk factors such as diabetes mellitus, fat intake, alcohol and coffee consumption, and smoking, confirmed risk factors remain unclear except for smoking.

In addition to an individual's lifestyle and medical history, geographical factors related to differences among countries have been investigated in regard to the occurrence of malignant neoplasm [10]. Several reports have suggested that sunlight exposure could influence the occurrence or prognosis of malignant neoplasm [11-13]. Kato et al. [14] reported that mortality from malignant neoplasm of the pancreas tends to be higher in northern Japan and in Scandinavian and other northern European countries, and that a strong positive correlation exists between mortality and latitude within Japan (correlation coefficients were 0.612 for males and 0.615 for females). International comparisons showed that mortality was also positively related to latitude (correlation coefficients were 0.724 for males and 0.725 for females). The average temperature was negatively correlated with mortality from malignant neoplasm of the pancreas within Japan (correlation coefficients were -0.587 for males and -0.630 for females), and a similar relationship was observed on an international scale (correlation coefficients were -0.773 for males and -0.729 for females). The change in latitude is thought to influence climatic factors such as sunlight and temperature. Mizoue [15] showed that exposure to solar radiation reduced the risk of malignant neoplasm of digestive organs, including the pancreas, and reported correlation coefficients between solar radiation and mortality from malignant neoplasm of the pancreas in Japan of -0.51 (p < 0.01) for males and -0.32 (p < 0.05) for females. To date, few reports have appeared concerning the influence of climatic factors such as sunlight exposure and temperature on malignant neoplasm of the pancreas. The purpose of this study is to examine the relationship between mortality from malignant neoplasm of the pancreas and climatic factors in Japan.

## Results

**Figure 1:**
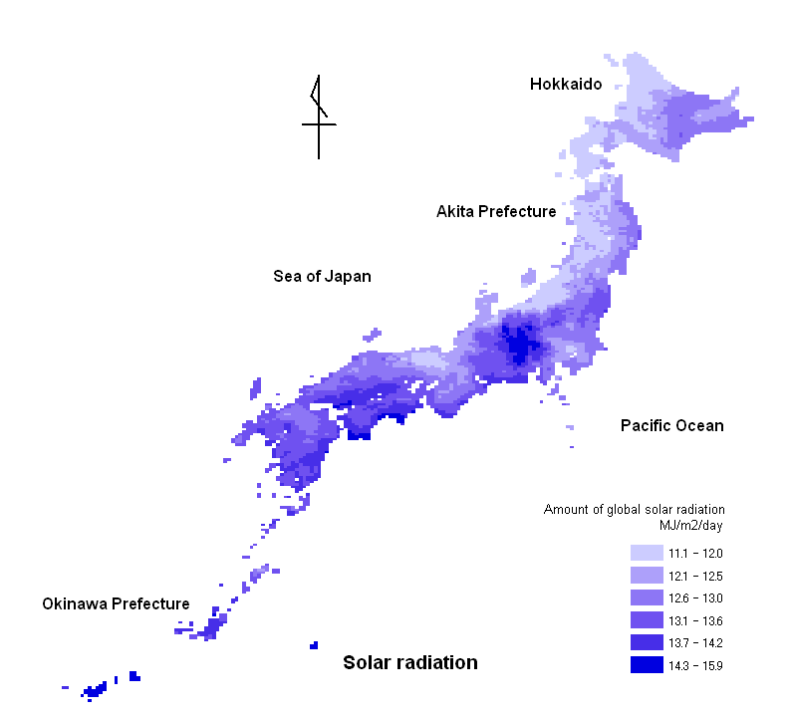
**Distribution map of amount of global solar radiation from 1971 to 2000 in Japan**. The map consists of 4,691 10-km mesh areas. The blue fill gradient indicates the amount of global solar radiation, with darker regions having a higher radiation level.

Figure 1 shows a distribution map of the amount of global solar radiation, consisting of 4,691 ten-kilometer (10-km) mesh areas. The amount of global solar radiation is the mean value of daily-accumulated solar radiation from 1971 to 2000 in Japan. The amount of global solar radiation tended to be low in northern areas along the Sea of Japan. 

**Figure 2:**
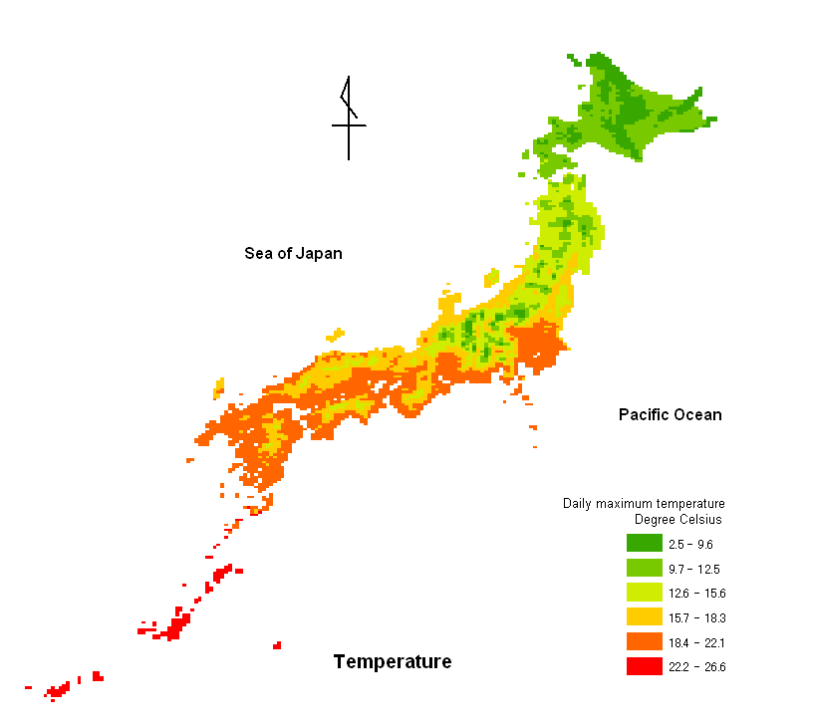
**Distribution map of mean daily maximum temperature from 1971 to 2000 in Japan**. The map consists of 4,691 10-km mesh areas. The fill in the mesh areas indicates daily maximum temperature, with green regions having a lower temperature, yellow and olive regions having a medium temperature, and red regions having a higher temperature.

Figure 2 shows a distribution map of the mean daily maximum temperature from 1971 to 2000 in Japan, consisting of 4,691 10-km mesh areas. The daily maximum temperature tended to be low in northern Japan.

**Figure 3:**
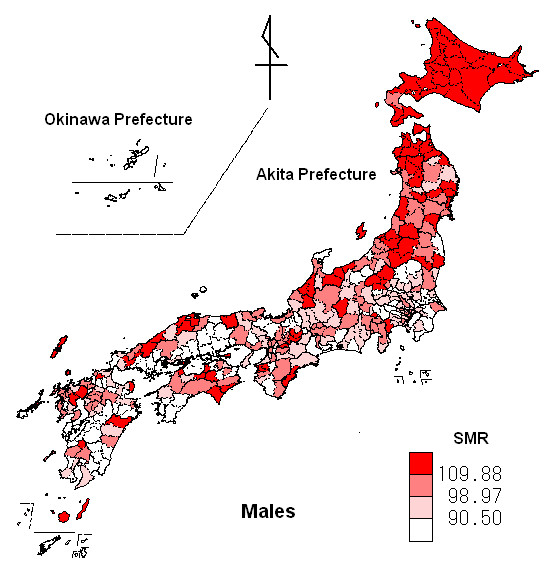
**Map of SMR for malignant neoplasm of the pancreas for males in Japan**. The red fill gradient indicates the standardized mortality ratio (SMR) of males for malignant neoplasm of the pancreas, with darker regions having a higher SMR. Black lines represent boundaries in SMR for malignant neoplasm of the pancreas for the second medical district in Japan.

**Figure 4:**
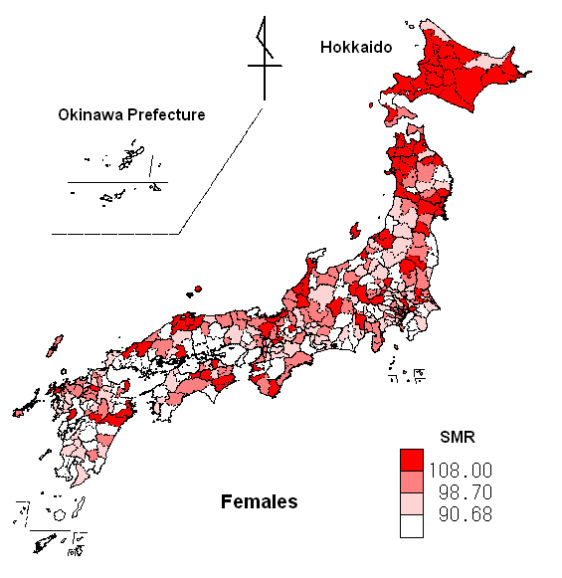
**Map of SMR for malignant neoplasm of the pancreas for females in Japan**. The red fill gradient indicates the SMR of females for malignant neoplasm of the pancreas, with darker regions having a higher SMR. Black lines represent boundaries in SMR for malignant neoplasm of the pancreas for the second medical district in Japan.

Figures 3 and 4 show disease distribution maps of standardized mortality ratio (SMR) for malignant neoplasm of the pancreas by second medical districts, using the choropleth method for males and females respectively. The SMR data were divided into 4 classes by quartile point. Northern Japan tended to show a high risk of development of malignant neoplasm of the pancreas in both males and females. 

Table 1 shows the basic statistics for SMRs for malignant neoplasm of the pancreas for 360 second medical districts for males and females, together with the amount of global solar radiation and daily maximum temperature. An SMR value of 100.00 indicates that the observed number of death from malignant neoplasm of the pancreas is the same as the expected number. The prefectures including the second medical districts with higher SMRs and the highest SMR are located in northern Japan (males: the highest SMR = 148.86 in Akita Prefecture; females: the highest SMR = 151.45 in Hokkaido) and those with lower SMRs and the lowest SMR are located in southern Japan (males: the lowest SMR = 43.25 in Okinawa Prefecture; females: the lowest SMR = 45.02 in Okinawa Prefecture). These trends were observed for both males and females.

**Table 1:** Basic statistics for SMRs for malignant neoplasm of the pancreas and climatic variables.

	Standardized mortality ratio (male)	Standardized mortality ratio (female)	Amount of global solar radiation ^a^(MJ/m^2^/day) ^b^	Daily maximum temperature ^c^(Degree Celsius)
Maximum	148.86	151.45	14.83	26.40
Minimum	43.25	45.02	11.53	7.84
Mean	100.95	100.05	12.89	17.29
Median	98.97	98.70	12.84	18.24
Number of sample	360	360	360	360

Amount of global solar radiation ^a^: Mean value of daily-accumulated solar radiation from 1971 to 2000 in Japan.

MJ/m^2^/day ^b^: Megajoule per square meter per day.

Daily maximum temperature ^c^: Mean value of daily maximum temperature from 1971 to 2000 in Japan. Standardized mortality ratios (SMRs) were calculated for 360 second medical districts.

Table 2 shows the Pearson's correlation coefficients of variables used in the analyses. SMRs for malignant neoplasm of the pancreas negatively correlated to the amount of global solar radiation and daily maximum temperature. The Pearson's correlation coefficients between solar radiation and SMR were -0.36 (p < 0.0001) for males and -0.34 (p < 0.0001) for females; daily maximum temperature and SMR were -0.57 (p < 0.0001) for males and -0.43 (p < 0.0001) for females.

**Table 2:** Pearson's correlation coefficients of variables used in the analyses.

	Standardized mortality ratio (male)	Standardized mortality ratio (female)	Amount of global solar radiation ^a^(MJ/m^2^/day) ^b^	Daily maximum temperature ^c^(Degree Celsius)
Standardized mortality ratio (male)	1			
Standardized mortality ratio (female)	0.46**	1		
Amount of global solar radiation ^a^(MJ/m^2^/day) ^b^	-0.36**	-0.34**	1	
Daily maximum temperature ^c^(Degree Celsius)	-0.57**	-0.43**	0.38**	1

Amount of global solar radiation ^a^:Mean value of daily-accumulated solar radiation from 1971 to 2000 in Japan.

MJ/m^2^/day ^b^: Megajoule per square meter per day.

Daily maximum temperature ^c^: Mean value of daily maximum temperature from 1971 to 2000 in Japan.

**: p ≦ 0.01

Tables 3 and 4 show the results of multiple linear regression analyses relating to SMRs for malignant neoplasm of the pancreas in males and females respectively. The regression coefficients for the amount of global solar radiation and the daily maximum temperature in males were -4.35 (p = 0.00034) and -2.81 (p < 0.0001) respectively. In females, the regression coefficients for the amount of global solar radiation and the daily maximum temperature were -5.02 (p < 0.0001) and -1.87 (p < 0.0001) respectively. The above results indicate that the SMR for males decreases by 4.35 with an increase in the amount of global solar radiation of 1 megajoule per square meter per day (MJ/m^2^/day) after taking into account the effect of daily maximum temperature. Similarly, the SMR for males decreases by 2.81 with an increase in the daily maximum temperature of 1 degree Celsius (°C) after taking the effects of solar radiation into account. The SMR for females decreases by 5.02 with an increase in the amount of global solar radiation of 1 MJ/m^2^/day after taking into account the effect of daily maximum temperature. The SMR for females decreases by 1.87 with an increase in the daily maximum temperature of 1°C after taking into account the effect of solar radiation.

**Table 3:** Regression coefficients for climatic factors on SMR for malignant neoplasm of the pancreas in males.

	Regression coefficient	Standard Error	p value
Amount of global solar radiation ^a ^(MJ/m^2^/day)^b^	-4.35	1.20	0.00034
Daily maximum temperature^c^(Degree Celisius)	-2.81	0.26	< 0.0001

Amount of global solar radiation ^a^: Mean value of daily-accumulated solar radiation from 1971 to 2000 in Japan.

MJ/m^2^/day ^b^: Megajoule per square meter per day.

Daily maximum temperature ^c^: Mean value of daily maximum temperature from 1971 to 2000 in Japan.

**Table 4:** Regression coefficients for climatic factors on SMR for malignant neoplasm of the pancreas in females.

	Regression coefficient	Standard Error	p value
Amount of global solar radiation ^a ^(MJ/m^2^/day)^b^	-5.02	1.25	0.000068
Daily maximum temperature ^c ^(Degree Celisius)	-1.87	0.27	< 0.0001

Amount of global solar radiation ^a^: Mean value of daily-accumulated solar radiation from 1971 to 2000 in Japan.

MJ/m^2^/day ^b^: Megajoule per square meter per day.

Daily maximum temperature ^c^: Mean value of daily maximum temperature from 1971 to 2000 in Japan.

## Discussion

The results of this study suggest that increasing solar radiation or temperature might decrease mortality from malignant neoplasm of the pancreas. Although excessive exposure to ultraviolet (UV) light may be harmful to our health (e.g. skin cancer), moderate exposure helps with the production of vitamin D. This ecological study indicated that solar radiation could be beneficial in preventing the development of malignant neoplasm of the pancreas. These results support previous reports for other malignant neoplasms, such as those involving the breast, colon and prostate [11,12]. Boscoe FP et al. [16] examined the relationship between ultraviolet -B (UV-B) exposure and 32 different cancer sites in the continental United States, and showed an evidence for an inverse association between mortality from malignant neoplasm of the pancreas and UV-B exposure for males and females. The relative risks of mortality related to solar UV-B exposure between the northern and southern United States boundary were 1.06 (95% confidence interval (CI), 1.03–1.09) for males and 1.11 (95% CI, 1.08–1.14) for females. Japan is located at a latitude similar to the continental United States and an inverse association between the solar radiation level and mortality from malignant neoplasm of the pancreas was observed, as seen in the continental United States [16]. The solar radiation levels of the Japanese Archipelago, however, do not show the obvious north-south gradient (UV-B level in the north is lower than that in the south) seen in the continental United States. The land features of Japan differ from those of the continental United States, being latitudinally long and thin, and include mountain ranges that run latitudinally through the Japanese Archipelago. Due to the land features of the Japanese Archipelago, climatic conditions differ between areas along the Sea of Japan and those occurring on the Pacific side of the archipelago. Since the climate in areas along the Sea of Japan tends to be influenced by the climate of the Eurasian continent, there are more rainy or snowy days in areas along the Sea of Japan than in areas of the Pacific side. Therefore, solar radiation levels for areas of the Japanese Archipelago adjoining the Sea of Japan tend to be lower than other areas. Figure 1 shows a distribution map of the amount of global solar radiation and depicts the situation described previously. The distribution map of standardized mortality ratios (SMRs) for males (Fig. 3) also shows that people living in areas of the Japanese Archipelago adjoining the Sea of Japan tend to be at higher risk. We investigated the relationship between mortality and solar radiation level (in the bottom and top quartiles). Mean SMR values were significantly higher in areas of low solar radiation, being 111.31 (95% CI, 107.63–114.98) for males and 107.08 (103.99–110.17) for females, when compared to areas of high solar radiation, which exhibited values of 94.49 (95% CI, 90.78–98.20) for males and 92.52 (89.10–95.94) for females. The above results concerning solar radiation were consistent with the study by Boscoe FP et al. [16]. Grant [17,18] reported the beneficial role of UV-B in the synthesis of vitamin D and concluded that UV-B radiation might be associated with a reduced risk of malignant neoplasms. Grant [18] also reported that these associations persisted even after adjustments were made for risk factors and risk reduction factors such as smoking and dietary intake.

Hokkaido, which is located in northern Japan, is thought to be a particularly high-risk area for males, since 20 of its 21 second medical districts possessed SMRs over the 0.75 quartile point of SMRs for males. Northern Japan also represents a high-risk area for females, although risk areas for females did not form a clear cluster. In our previous study in the Ibaraki Prefecture, we observed geographical clustering of SMRs for males but not for females [19].

SMR values lack stability in thinly populated areas, such as small towns or villages. In this study, we calculated the SMRs for second medical districts that consist of several municipalities whose population is roughly 100,000 to 200,000. The instability of SMRs in small populations may thus be somewhat reduced by this process.

We also found that SMRs for malignant neoplasm of the pancreas tended to be low in temperate regions. Mean SMR values were significantly higher in areas of low maximum temperature, being 115.31 (95% CI, 111.65–118.98) for males and 108.54 (104.74–112.35) for females, when compared to areas of high maximum temperature, which exhibited values of 93.01 (95% CI, 90.25–95.77) for males and 93.18 (90.22–96.13) for females. The mean daily maximum temperature in Hokkaido (northern Japan) was obviously lower than that of other areas (Fig. 2). The distribution maps of SMR showed that Hokkaido was at higher risk for males and females (Figs. 3 and 4). The impact of low temperature may play a more important role than low solar radiation for the risk of malignant neoplasm of the pancreas. Although few papers describe the relationship between temperature and malignant neoplasm, Laskin et al. [20] reported that ultraviolet light and temperature influenced cell proliferation. Holick [21] conducted an animal study and showed that the transformation of previtamin D_3 _to vitamin D_3 _depended on temperature, and that the process of transformation was more enhanced at 25°C than at 5°C. There is increasing evidence that seasonal variation is observed for the prognosis of several cancers [22][23][24][25]. Porojnicu AC et al. reported that reliable prognoses were seen for summer and autumn diagnoses, corresponding to maximal calcidiol levels for colon cancer, prostate cancer, breast cancer, Hodgkin lymphoma and lung cancer [22][23][24]. They also reported that skin synthesis of vitamin D_3 _by solar radiation was reduced during winter in Norway due to insufficient sun exposure during this season [23]. Since an animal study has shown that the synthesis of vitamin D_3 _is enhanced at higher temperatures [21], low temperatures during winter may reduce the synthesis and action of vitamin D_3 _in the human body. In addition to solar radiation, temperature may influence the development of malignant neoplasm of the pancreas. The impact of climatic conditions on the human body may vary due to individual lifestyle or occupation. Since SMRs and mesh climatic data are based on group data and not individual data, the impact of climatic conditions on the development of malignant neoplasm of the pancreas may not have been predicted accurately in this study. Mesh climatic data, however, might prove useful as indices of climatic factors that affect the human body.

This study was conducted only within Japan. Colli JL et al. [26] made international comparisons regarding the effect of ambient sunlight exposure and consumption of foods on prostate cancer mortality, and showed that sunlight level was inversely associated with prostate cancer mortality. Though their results were consistent with other studies, the strength of the association was weaker (p = 0.001) than that of other studies. There was wide variation in lifestyle and diets for inhabitants of countries represented in the international study. They found a strong correlation between the consumption of cereals and reduced prostate cancer risk. When people move or emigrate to another place, the residential environment changes but their lifestyle or dietary pattern may not change significantly. Severson RK et al. [27] reported an association between diet and prostate cancer for Japanese immigrants in Hawaii. The prostate cancer risk for Japanese living in Hawaii is higher than that for Japanese living in Japan, although it is lower than the risk for Hawaiian whites. Their study found that the lower risk of prostate cancer for Japanese living in Hawaii relative to Hawaiian whites was associated with the consumption of rice. In a similar way, the pancreas is a digestive organ that secretes pancreatic juice, and dietary habits may have an important effect on the risk of the development of malignant neoplasm.

In this study, we used mortality data from 1998 to 2002. Since the latency period of malignant neoplasm can be 20 to 30 years following exposure to a risk factor, the data-collecting period regarding smoking status should be from 1970 to 1980. However, surveys conducted by the Ministry of Health, Labour and Welfare that pertain to smoking began in 2001. The data-collecting period on smoking status is the same as that concerning mortality data. Consequently, we did not make an adjustment of SMRs for smoking status, though smoking status appears to be an important factor affecting the occurrence of malignant neoplasm of the pancreas [2]. Smoking, diabetes mellitus and fat intake are suspected risk factors for malignant neoplasm of the pancreas [2,4,8]. In a previous study, Kinoshita S [28] reported that mortality from malignant neoplasm of the pancreas was inversely associated with solar radiation level and temperature. Even after adjustments were made for suspected risk factors such as smoking, diabetes mellitus and fat intake, the aforementioned association persisted. We hope to further examine this phenomenon in the future using consecutive detailed surveys on smoking status in Japan.

Further investigation is required to identify the association between climatic factors and malignant neoplasm of the pancreas. In Japan, over twenty thousand people die annually of malignant neoplasm of the pancreas. We recommend additional studies using individual data to evaluate the impact of environmental factors on the human body in order to facilitate the primary prevention of this disease. We would like to conduct further studies using individual data and to examine the effect of climatic factors on the development of malignant neoplasm of the pancreas with making adjustment for suspected risk factors.

## Conclusion

This paper explores the relationship between malignant neoplasm of the pancreas and climatic factors, such as the amount of global solar radiation and daily maximum temperature, using mesh climatic data for Japan. Increases in solar radiation and temperature were significantly related to a decrease in SMRs for malignant neoplasm of the pancreas in both males and females.

The disease distribution maps using SMRs for malignant neoplasm of the pancreas showed that males and females in northern Japan tend to be at higher risk. The distribution map for solar radiation showed that solar radiation in northern Japanese areas along the Sea of Japan tends to be low. Daily maximum temperature is obviously low in northern Japan.

The effect of climatic factors on SMRs for malignant neoplasm of the pancreas may be summarized as follows: 1 MJ/m^2^/day of solar radiation can change the SMR for malignant neoplasm of the pancreas by -4.35 and -5.02 in males and females, respectively, after taking into account the effect of daily maximum temperature; and an increase of 1°C in daily maximum temperature can change the SMR for malignant neoplasm of the pancreas by -2.81 and -1.87 in males and females, respectively, after taking into account the effect of solar radiation.

## Methods

### Medical data

The number of deaths from malignant neoplasm of the pancreas (Complying with the International Classification of Disease, tenth revision (ICD-10): C25) categorized by sex and age for each municipality and total for Japan were obtained from the National Vital Statistics database from 1998 to 2002 (the Ministry of Health, Labour and Welfare. Vital Statistics on Deaths of Japan). The data on population categorized by sex and age for each municipality were obtained from the 2000 Population Census by the Statistics Bureau, Ministry of Internal Affairs and Communications. The data on total population from 1998 to 2002 for Japan was obtained from the Population Estimates by sex and age (5-year group) by the Statistics Bureau, Ministry of Internal Affairs and Communications. The list of the second medical districts with municipalities in 2000 was obtained from the hospital report by the Ministry of Health, Labour and Welfare (National health statistics 2001). Municipalities were joined according to the list of medical district in 2000. The second medical districts that are defined roughly as consisting of 100,000 to 200,000 for populations and 500 to 1,000 square kilometer (km^2^) for land areas, are divided by the boundary of livelihood sphere for residents. A second medical district-based partition may be more effective to explore the risk of the development of malignant neoplasm rather than a prefecture-based partition since environmental factors may be similar within the same livelihood sphere. Miyake Island was omitted since the population of Miyake Island in Tokyo in 2000 was zero given complete evacuation of the island due to volcanic activity. We obtained the boundary files for municipalities in 2000 from the Geographical survey institute. Standardized mortality ratio (SMR) for each second medical district was calculated using total Japan as a reference population.

### Meteorological data

Mesh climatic data for Japan published in 2006 were obtained from the Japan Meteorological Agency. The data included temperature (mean, maximum, and minimum temperature), global solar radiation, sunshine, precipitation, and snow cover. Mesh climatic data are the estimated values for a one-kilometer (1-km) mesh, estimated by the average of observations made from 1971 to 2000. Data for the amount of global solar radiation and the daily maximum temperature were used for analyses. Since the file size of 1-km mesh data throughout Japan was too large for us to computerize, we used ten-kilometer (10-km) mesh data as average values, calculated from the 1-km mesh data.

### Data analyses

A linear regression model was employed to predict SMR for malignant neoplasm of the pancreas in relation to changes in solar radiation and daily maximum temperature. The following model was used for this analysis:

Ŷ_[i] _= k_0_+ k_1 _X_1 [i] _+ k_2 _X_2 [i]_

where Ŷ is the linear predictor of SMR for malignant neoplasm of the pancreas in area [i], X_1 _is the amount of global solar radiation in area [i], X_2 _is the daily maximum temperature in area [i], and k_1 _and k_2 _are the regression coefficients that respectively represent the effect of X_1 _and X_2 _on Ŷ.

'R version 2.2.1' (R Foundation for Statistical Computing, Vienna, Austria) was used for the statistical analysis. 'MANDARA version 7.25' (1992–2006 Tani Kenji, Saitama, Japan), which is the software of the geographical information analysis support system, was used for drawing disease maps. 'ArcView 9.1' (Environmental Systems Research Institute, Inc. (ESRI)) was employed for drawing distribution maps for climatic factors.

## Competing interests

The author(s) declare that they have no competing interests.

## Authors' contributions

Authors SK, YW and MO collaborated intensely on all aspects of the manuscript: research design, data preparation, statistical analysis, and discussion. All authors read and approved the final manuscript.
